# Decoding the contents and strength of imagery before volitional engagement

**DOI:** 10.1038/s41598-019-39813-y

**Published:** 2019-03-05

**Authors:** Roger Koenig-Robert, Joel Pearson

**Affiliations:** School of Psychology, The University of New South Wales, Sydney, Australia

## Abstract

Is it possible to predict the freely chosen content of voluntary imagery from prior neural signals? Here we show that the content and strength of future voluntary imagery can be decoded from activity patterns in visual and frontal areas well before participants engage in voluntary imagery. Participants freely chose which of two images to imagine. Using functional magnetic resonance (fMRI) and multi-voxel pattern analysis, we decoded imagery content as far as 11 seconds before the voluntary decision, in visual, frontal and subcortical areas. Decoding in visual areas in addition to perception-imagery generalization suggested that predictive patterns correspond to visual representations. Importantly, activity patterns in the primary visual cortex (V1) from before the decision, predicted future imagery vividness. Our results suggest that the contents and strength of mental imagery are influenced by sensory-like neural representations that emerge spontaneously before volition.

## Introduction

A large amount of psychology and, more recently, neuroscience has been dedicated to examining the origins, dynamics and categories of thoughts^1–3^. Sometimes, thoughts feel spontaneous and even surprising; while other times they feel effortful, controlled and goal oriented. When we decide to think about something, how much of that thought is biased by pre-existent neural activity? Mental imagery, a sensory thought, can be triggered voluntarily or involuntarily^4^. However, how much of the content and strength of our mental images we actually control when we voluntarily generate imagery remains unknown. For example, individuals with post-traumatic stress disorder (PTSD) report a complete lack of control of both the content and strength of their mental imagery^5^. While evidence suggests that imagery strength varies both between and within individuals in the normal population^5,6^. Previous research has shown that prefrontal activity can predict future decisions^7–10^, and nonconscious sensory activity^11^, and that mental images can be decoded from early visual cortex^12,13^. However, it remains unknown whether nonconscious sensory activity influences what we think and how strongly we think it.

To investigate the origins of the content and strength of voluntary imagery, we crafted a thought-based mental imagery decision task, in which individuals could freely decide what to imagine, while we recorded brain activation using functional magnetic resonance imaging (fMRI). We used multi-voxel pattern analysis (MVPA, see Materials and Methods for details) to decode information contained in spatial patterns of brain activation recorded using fMRI^14–16^. Additionally, in an independent control experiment, we estimated the temporal reliability of the reported onset of thoughts, as it has been criticized in previous paradigms^17^. Using a design exploiting the known effect of imagery priming on subsequent binocular rivalry as a function of time^18^, we show that participants’ reports of thought onsets were indeed reliable within the temporal resolution of fMRI.

Models of determinants of decision making postulate that executive areas in the prefrontal cortex would trigger selection processes leading to future choices^9,10,19^. In addition to the executive areas involvement in future visual thoughts, we aimed to test whether predictive information could also be decoded from visual areas, as previous results have shown that visual imagery recruits visual areas^12,13^. To test this, we used both searchlight and visual (from V1 to V4) regions-of-interest (ROI) decoding. We also sought to determine the representational content of the predictive signals: is predictive information, to some extent, similar to perceptual visual representations? To assess this, we perceptually presented gratings outside of attention to participants in separate runs. Functional brain images from the perceptual blocks were then used to train classifiers, which were subsequently tested on imagery blocks both before and after the decision. This so called perception-imagery generalization cross decoding was thus used to show common informational content between visual perceptual representations and predictive signals. Finally, we tested whether the subjective strength of visual imagery could be decoded from information in visual areas before reported volition. Such an involvement of visual areas in the future strength of visual imagery would provide further evidence that sensory areas also play an important role in the phenomenology of future thoughts.

Using this paradigm, we found that activity patterns were predictive of mental imagery content as far back as 11 seconds before the voluntary decision of what to imagine –in visual, frontal and subcortical areas. Importantly, predictive patterns in the primary visual cortex (V1) and the lateral prefrontal cortex were similar to perceptual representations elicited by unattended images. We show that the subjective strength (vividness) of future mental imagery can be predicted from activation patterns contained in the primary visual cortex (V1) before a decision is made. Our results suggest that the contents and strength of mental imagery are influenced by sensory-like neural representations that emerge spontaneously before volition. These results are important as they point to a role of visual areas in the pre-volitional processes leading to visual thought production, thus shedding light on the mechanisms of intrusive mental imagery in conditions such as PTSD, as well as the origins of normal mental imagery.

## Results

### Free decision visual imagery paradigm

**Figure 1:**
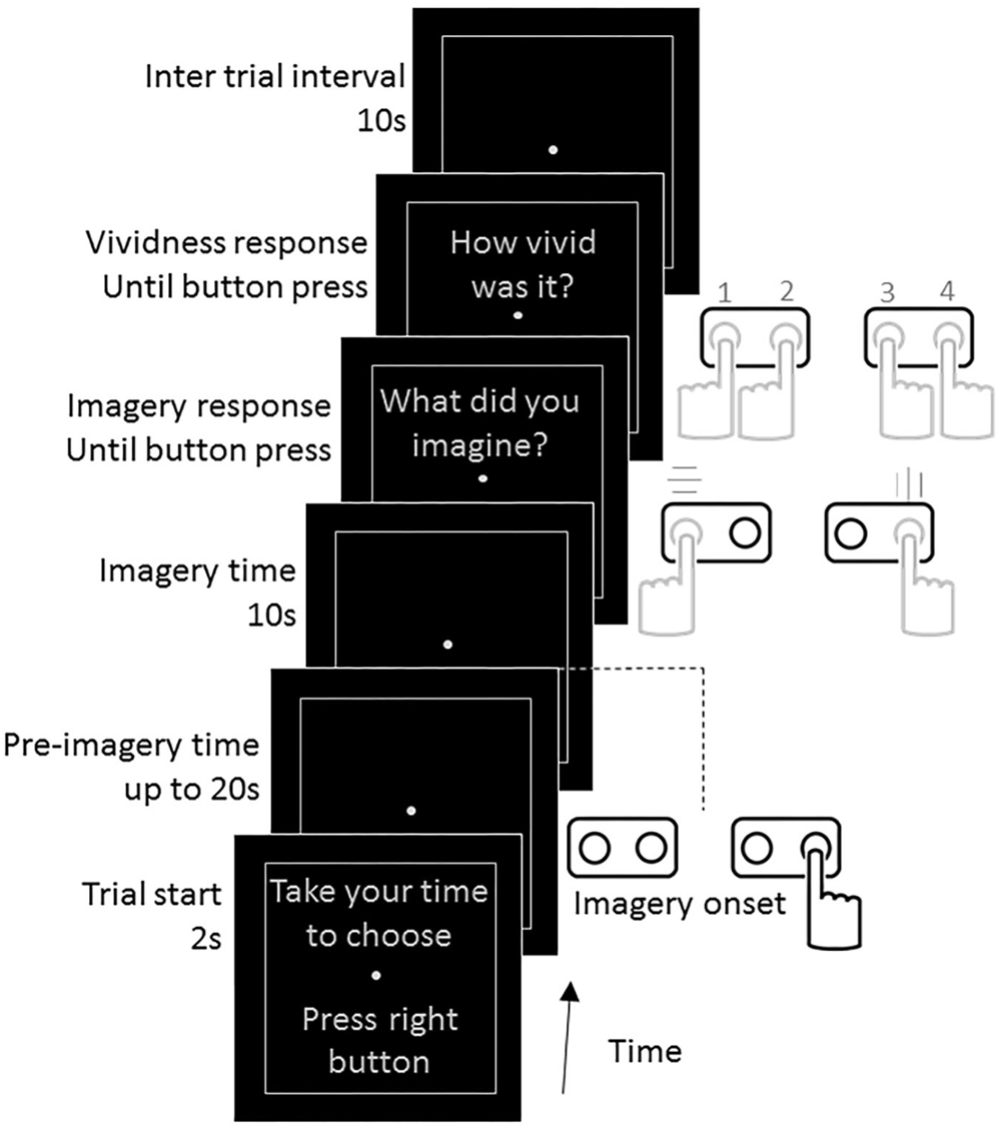
fMRI task paradigm. Participants had to freely choose between two predefined gratings (horizontal green/vertical red or vertical green/horizontal red, counterbalanced across participants). Each trial started with the prompt: “take your time to choose – press right button” for 2 seconds. While the decision was made, a screen containing a fixation point inside a rectangle was shown. This period is referred as “pre-imagery time” and was limited to 20 seconds. Participants were instructed to press a button with the right hand as soon they decided which grating to imagine (always the same button independently of the chosen grating). During the imagery period (10 seconds), participants imagined the chosen grating as vividly as possible. At the end of the imagery period, a question appeared on the screen: “what did you imagine? – Left for vertical green/red – Right for horizontal red/green” (depending on the pre-assigned gratings for the participant). After pressing the relevant button to answer, a second question appeared: “how vivid was it? –1 (low) to 4 (high)”, to which participants answered using one of 4 buttons. After each trial there was a blank interval of 10 seconds where we instructed the participants to relax and not to think about the gratings nor subsequent decisions. Gray hand drawings represent multiple possible button responses, while black drawing represents a unique button choice.

Our paradigm consisted of a mental decision leading to the formation of a visual mental image. In every trial, participants had to choose to imagine one of two possible different colored and oriented gratings while we recorded brain blood-oxygen-level dependent (BOLD) using fMRI (Fig. 1, see Materials and Methods for details). After the start of the trial, participants had a maximum of 20 seconds to freely decide which pattern to think of. As soon as they felt they had made the decision, they pressed a button (always the same button for both gratings) with the right hand, thus starting 10 seconds of imagery generation. During this time, participants imagined the chosen grating as vividly as they could. Subsequently, they were prompted with two questions: “what did you imagine?” and “how vivid was it”, to which they answered by pressing different buttons (Fig. 1). On average, participants took 5.48 s (±0.15 SEM) to decide which grating to imagine, while the average trial time was 31.18 s (see Fig. S1 and Materials and Methods for details). Each trial included a blank period of 10 s at the end to avoid spillover effects from one trial to the next^20,21^. Participants chose to imagine each grating with similar probabilities (50.44% versus 49.56% for vertical and horizontal respectively, Shannon entropy = 0.997, with a switch probability of 58.59% ±2.81 SEM, see Materials and Methods for detailed behavioral results).

### Decoding sanity checks

We first verified the suitability of our decoding approach to classify the contents of visual perception and imagery. We used SVM classifiers trained and tested (in a cross-validation scheme) on 10 s of perception or imagery data and classified the perceptual or imagined stimuli (red/green horizontal/vertical gratings) in visual areas from V1 to V4 (see Materials and Methods for details). Fig. S2 shows the results of this sanity check. We found above chance decoding accuracy for perception (91.7, 91.7, 91.7 and 71.4%; one-tailed t-test p = 3.1·10^−8^, 1.2·10^−9^, 7·10^−11^ and 1.5·10^−3^; from V1 to V4) and imagery (66.9, 67, 69.1 and 63.7%; p = 8·10^−4^, 1.2·10^−3^, 1·10^−4^ and 8·10^−3^). These results are comparable to previous results on decoding perception and imagery^22–24^ and thus validate our decoding approach.

### Searchlight decoding results

To investigate which brain areas contained information about the contents of imagery, we employed a searchlight decoding analysis on fMRI data from the whole brain^16^. We used two sources of information to decode the contents of imagery: neural activation patterns within the imagery condition (imagery decoding) and patterns from unattended perceptual stimuli to decode imagery data (perception-imagery generalization cross-decoding). For the imagery decoding, we trained and tested classifiers using the imagery data. In the imagery-perception generalization analysis we trained the classifiers using data from the perception scans and tested on imagery data. The latter allowed us to explore shared information between perception and imagery, without the effects of attention (see Materials and Methods for details & behavioral attention task during perception).

We defined the areas bearing information about the contents of imagery as those revealing above chance decoding accuracy at any point in time during a 28 s time window around the decision (cluster definition threshold p < 0.001, cluster threshold p < 0.05, see Materials and Methods for details). Under this selection criterion, above chance decoding at any point in time is trivial and not relevant for our question. Rather, the purpose of this analysis is investigating the temporal dynamics of the imagery-content information. Specifically, we were interested to test whether any area contained information about the contents of imagery before the decision. In this respect, our analysis is bias-free regarding the temporal position of the information, as we considered many time-points before and after the decision (7 points each).

**Figure 2:**
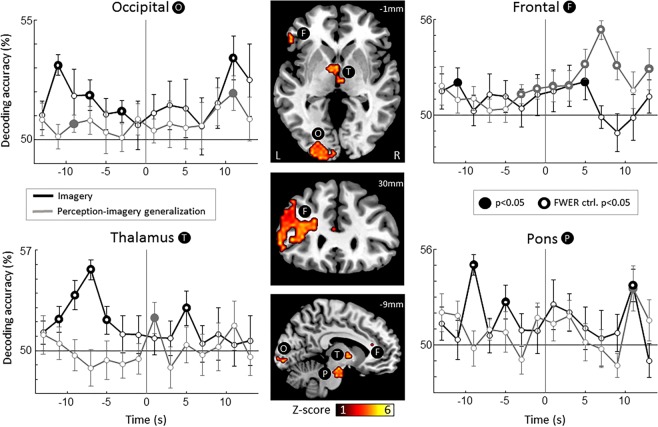
Searchlight decoding of the contents of imagery. Using searchlight decoding, we investigated which regions contained information about the contents of mental imagery (see Materials and Methods for details). We defined these regions as those showing above chance accuracy at any point in time (Gaussian random field correction for multiple comparisons, see Materials and Methods for details). We found 4 such regions (central panels): occipital (O), frontal (F), thalamus (T) and pons (P). Then, we investigated the temporal dynamics of each one of these regions (lateral plots), from −13 to +13 seconds from the voluntary imagery onset (time = 0). We decoded imagery contents using the information from imagery runs (imagery, black line) and using information from perception (perception-imagery generalization, grey line). For the imagery decoding (black line), all four regions showed significant above-chance accuracy both before and after imagery onset, indicating that information from imagery was predictive of the chosen grating before (up to −11 seconds) and after the imagery onset. On the other hand, the perception-imagery generalization (grey line) showed significant above-chance decoding before the onset of imagery only in occipital and frontal areas, indicating that perceptual-like information was predictive of the chosen grating before the imagery onset only in cortical areas and after the imagery onset in both cortical and subcortical areas. Numbers on upper-right slices’ corners indicate MNI coordinates. Error bars represent SEM across participants. Full circles represent above chance decoding (p < 0.05, one-sample t-test against chance: 50%). White points inside full circles represent time courses where the number of significant points was significantly above chance level after correction for family-wise error rate (p < 0.05, permutation test, see Fig. S3 for details).

Using the above explained analysis, we found a network of four areas: frontal, occipital, thalamus and pons (Fig. 2, central panels, see Table S1 for cluster locations in MNI coordinates). We then examined the information-content time course in these areas from −13 to +13 seconds from the reported imagery decision (time = 0). As expected, time-resolved (2 s) decoding yielded lower (but statistically significant) accuracies than averaging over longer periods (see Fig. S2 for comparison), presumably due to its lower signal-to-noise ratio. Importantly, in the context of neuroscience research, decoding accuracy scores are not the most relevant output of classification, but rather their statistical significance is^25^. Time-resolved classification in the imagery condition reached above chance decoding accuracy up to 11 seconds before reported imagery onset in occipital and thalamus while significant classification was reached at −9 seconds in the pons (Fig. 2; black solid points with inner white circle, p < 0.05, one-sample, one-tailed t-test, controlled for FWER p < 0.05, permutation test, see methods for details).

**Figure 3:**
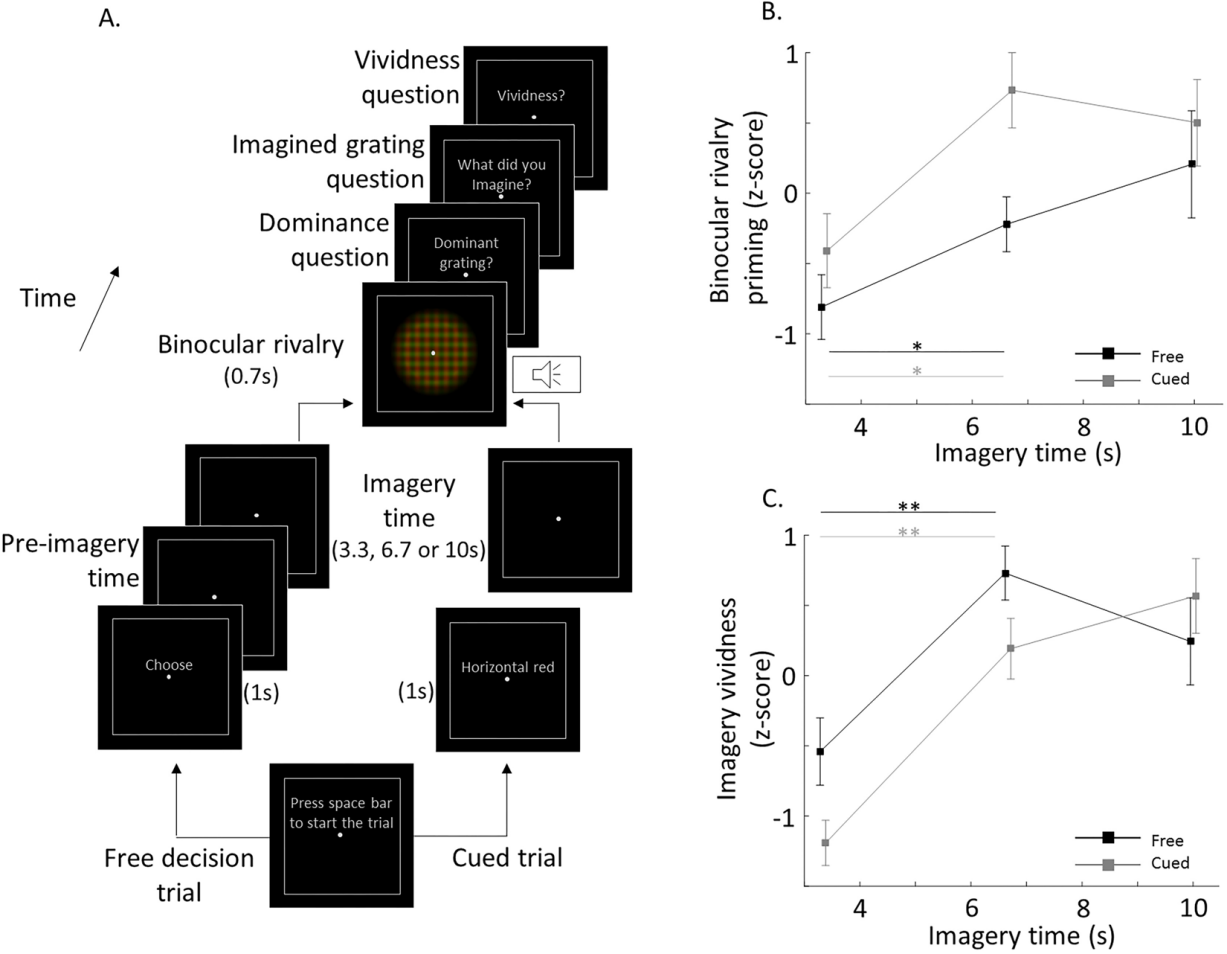
Behavioral experiment: testing the accuracy of imagery onset reports. We tested perceptual priming and subjective imagery vividness a function of imagery time as a means to verify the accuracy of reporting the imagery onset. (**A**) Paradigm. Free decision and cued trials were pseudo-randomized. Perceptual priming was measured as a function of imagery time (3.3, 6.7 and 10 s), as the dominance bias on binocular rivalry. (**B**) Perceptual priming. Imagery time significantly increased perceptual priming on the free decision and cued conditions (ANOVA, F = 7.15, p = 0.002), and priming in the free decision condition was significantly lower than in the cued condition (ANOVA, F = 5.77, p = 0.021), thus ruling out that participants were reporting the imagery onset after starting imagining. (**C**) Imagery vividness. Imagery time also significantly increased subjective imagery vividness on the free decision and cued conditions (ANOVA, F = 18.49, p < 10^−5^). Stars show significant differences between the first two time points, thus setting a lower bound of temporal resolution on this behavioral task. These results show that the reported onset of imagery is reliable relative to the temporal resolution of fMRI. Error bars show ±SEM. Black and gray lines present free and cued conditions, * and ** represent p < 0.05 and p < 0.01, two-sample t-test.

The perception-imagery generalization decoding showed significant above chance accuracy as early as −9 seconds before the onset of imagery in occipital areas (although these results did not survived the control for FWER) and −3 seconds in frontal areas (Fig. 2; grey solid points with inner white circle, p < 0.05, one-sample, one-tailed t-test, controlled for FWER p = 0.003, permutation test), indicating that pre-volitional predictive information shares properties with perception in frontal areas. In subcortical areas, above-chance generalization decoding accuracy was only observed after the onset of imagery (+1 and +11 seconds in the thalamus and the pons respectively) and was not significant after controlling for FWER. Importantly, during the perceptual scans visual attention was diverted by a demanding fixation task (see Materials and Methods), hence such generalization should not be due to high-level volitional or attentional mechanisms. Interestingly, decoding accuracy in occipital areas during the imagery period was lower than expected (see for example^26^). Previous studies have shown that prior decisions can impair subsequent cognitive tasks^27^. Therefore, the cognitive load for the decision element of our task could impair imagery, which is consistent with the results of a behavioral control experiment showing that cued imagery (no-decision) was stronger than decision followed by imagery (Fig. 3B,C).

### Behavioral imagery onset reliability experiment

We ran an independent behavioral experiment outside the scanner to test whether participants might have begun imagining before they reported having done so, which could explain early above chance classification. We utilized a method that exploits binocular rivalry to objectively measure imagery strength^18,28^ as a function of time in a free decision and a cued condition (Fig. 3). We reasoned that if participants were reporting the onset of imagery a few seconds late, this would be detected as an increase in rivalry ‘priming’ compared to a condition where the onset of imagery is controlled by the experimenter, as such priming is known to be dependent on time^18^. Figure 3B shows the effects of imagery time on sensory priming for both conditions. Imagery time showed a significant effect on priming for free decision and cued conditions (ANOVA, F = 7.15, p = 0.002, Fig. 3B), thus confirming the effect of imagery time on priming. Priming for the free decision condition was significantly lower than in the cued condition (ANOVA, F = 5.77, p = 0.021), indicating that participants did not start imagining before they reported doing so (which would have resulted in the opposite pattern) and also suggesting that sensory priming is somehow disrupted by the decision task, perhaps due to cognitive load, analogous to what has been shown in other cognitive tasks^27^. Importantly, significant differences in priming between 3.33 and 6.67 seconds of imagery time were found for the free decision and cued conditions (one-tailed t-test, p < 0.05), indicating that this behavioral task can resolve differences in priming spaced by 3.33 seconds, at least for these two first time points, thus providing a lower bound of temporal resolution of the accuracy of the reported imagery onset which is comparable to that of fMRI.

Figure 3C shows the effects of imagery time on subjective imagery vividness. Imagery time showed also a significant effect on vividness for free decision and cued conditions (ANOVA, F = 18.49, p < 10^−5^, Fig. 3C). However, differences between free decision and cued conditions were not significant (ANOVA, F = 2.42, p = 0.127). Again, significant differences in vividness between 3.33 and 6.67 seconds of imagery time were found for the free decision and cued conditions (one-tailed t-test, p < 0.01). While a similar pattern of results could arguably be explained by subjects starting to imagine the opposite target before they reported it, or imagining the two possible targets alternatively, these outcomes are not consistent with our fMRI results. This control largely overcomes one of the major limitations to prior free-choice paradigms, as it enables us to measure precision of thought-choice reporting^17^.

### Searchlight decoding control analyses

We employed a permutation test to check whether the decoding distributions contained any bias, in which case above chance decoding would be overestimated and the use of standard parametric statistical tests would be invalid^29^ (see Materials and Methods for details). Permutation tests yielded similar results to those using parametric tests (Fig. S4), and, importantly, decoding accuracy distributions under the null hypothesis showed no bias, thus validating the use of standard parametric statistical tests (Table S2).

We also conducted a control analysis to test whether the searchlight results could be explained by any spillover from the previous trial. We trained the classifiers on the previous trial (N-1 training) and tested on the subsequent trial (trial N). If there was spill over from the previous trial, this analysis should show similar or higher decoding accuracy in the pre-imagery period. We found no significant above chance classification for any of the regions, thus ruling out the possibility that these results are explained by any spill over (Fig. S5).

### Visual regions-of-interest (ROI) decoding

Results from the searchlight analysis were inconclusive regarding whether the predictive information before the decision share similarities with visual perception, as only frontal areas exhibited robust perception-imagery generalization decoding (Fig. 2). To test whether predictive information can be found in visual areas, we conducted a time-resolved decoding analysis only in visual regions-of-interest (ROI) from V1 to V4 defined by an independent functional experiment (see Materials and Methods for details). We reasoned that if we find information that predicts the imagery decision in perception-devoted visual areas this would be a strong argument in favor of perceptual predictive information.

**Figure 4:**
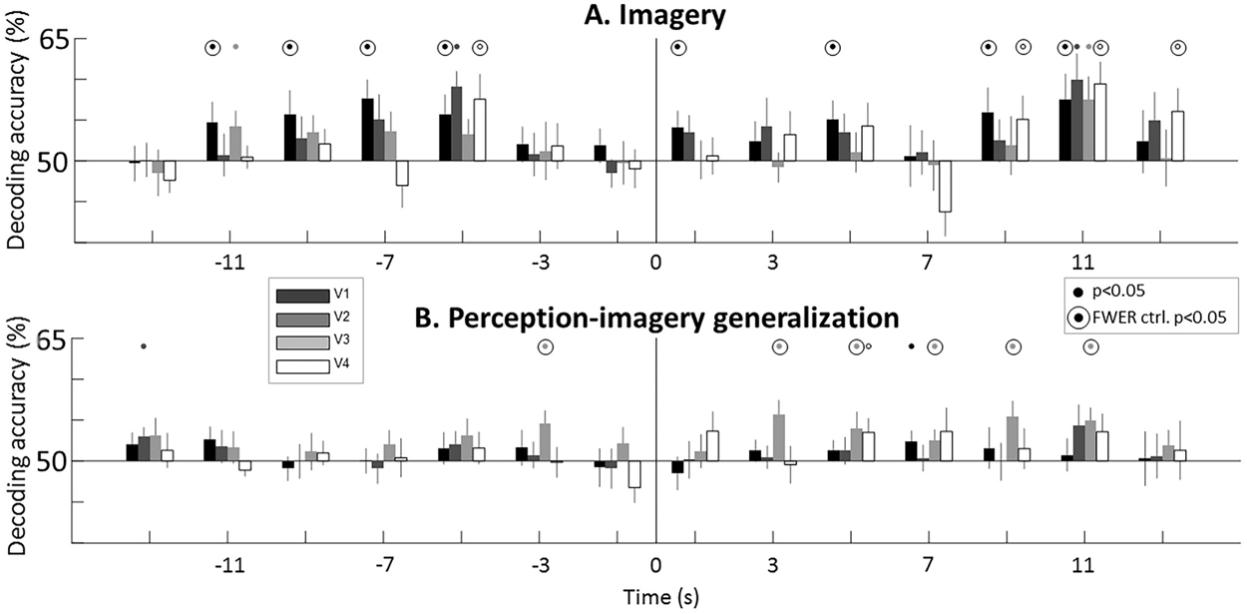
Decoding the contents of imagery in visual regions-of-interest (ROI). We examined the contents of imagery in visual areas using a ROI approach. Visual areas from V1 to V4 were functionally defined and restricted to the foveal representation (see Materials and Methods for details). (**A**) Imagery decoding. We found above-chance decoding accuracy for imagery decoding both before (from −11 seconds) and after imagery onset. Different visual ROI showed significant above-chance decoding accuracy at different time points, while V1 ROI was the most consistent across time points. (**B**) Perception-imagery generalization. The cross-decoding generalization analysis showed consistent above chance decoding accuracy only in V3. Error bars represent SEM across participants. Full points represent above chance decoding (p < 0.05, one-sample t-test against chance: 50%). Outline circles represent time courses where the number of significant points was significantly above chance level after correction for family-wise error rate (p < 0.05, permutation test, see Fig. S3 for details).

The imagery ROI decoding analysis revealed similar temporal dynamics to the searchlight approach, showing earliest above-chance decoding accuracy −11 seconds from the reported imagery decision in the primary visual cortex, V1 (Fig. 4A). In the imagery decoding, all visual ROIs showed above chance decoding accuracy before imagery onset at different time points (small points, Fig. 4A, p < 0.05, one-sample, one-tailed t-test against chance: 50%), however only V1 and V4 were consistent across time points (from −11 to −5 and to −5 to 15 seconds, Fig. 4A outline circles, p < 0.05, one-sample, one-tailed t-test, controlled for FWER p < 0.05, permutation test). The early (−11s) predictive information in primary visual cortex suggest that predictive signals would correspond, at least partly, to visual representations.

The perception-imagery generalization showed more modest effects with above chance decoding accuracy in V3 just 3 s before imagery onset (Fig. 4B outline circles, p < 0.05, one-sample t-test against chance: 50%, controlled for FWER p < 0.003, permutation test). The overall low perception-imagery generalization decoding accuracy after imagery onset suggests that the analysis might not be effectively capturing the representational commonalities between perception and imagery as reported previously^30,31^. This discrepancy with previous results could be due to experimental noise or to a lack of representational similarity between perception and imagery. To distinguish between these two alternatives, we performed a new analysis seeking more sensitivity by abandoning the time-resolved analysis as described in the next section.

### Predictive information in visual areas shares properties with perceptual information

**Figure 5:**
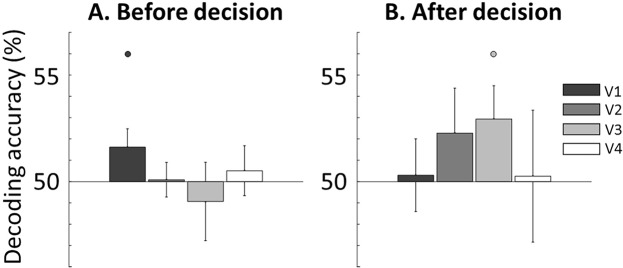
Predictive signals in visual areas share information with perceptual representations before and after the decision. In order to test whether predictive information in visual areas shared properties with perceptual representations in visual areas, we conducted generalization decoding by training on perception and testing on imagery data, during a period before (−10 to 0 s) and after the decision (0 to 10 s). Error bars represent SEM across participants. Significant decoding was found in V1 and V3 before and after the decision, respectively (p < 0.05, one-sample t-test against chance: 50%).

While imagery decoding in visual areas suggests that predictive information is perceptual in nature, it does not rule out other possibilities, such as attentional effects. In particular, the time-resolved generalization analysis failed at showing strong decoding before the decision in visual areas (Fig. 4A). This can be due to a number of factors such as differences in the neural representations between imagery and perception, as well as the differences in signal-to-noise ratio between these conditions, which could lead to poor classification performance. We thus tested whether abandoning the time-resolved analysis would produce more conclusive results by increasing the signal-to-noise ratio. To achieve more sensitivity, we trained classifiers on perception runs and tested them on the imagery before-decision period (−10 to 0 s) and the after-decision period (0 to 10 s), thus effectively pooling the data across time points for the imagery condition as opposed to analyzing each point separately (as it was done in the time-resolved analysis, Fig. 4B). This analysis showed modest but significant decoding before the decision in V1, and after the decision in V3 (Fig. 5, solid points, p < 0.05, one-sample, one-tailed t-test against chance: 50%). This result thus supports the idea that predictive information is at least partly perceptual in nature and that the predictive perceptual representations would be housed in the primary visual cortex.

### Visual areas ROI decoding control analyses

We also conducted a number of control tests on the ROI decoding results to ascertain the validity of our results. Permutation tests on the ROI decoding yielded similar results (Fig. S6). We controlled for whether these results could be accounted by any spill over from previous trials by again conducting an N-1 analysis. This analysis did not show any above chance accuracy before the imagery onset, but we found a significant time point at t = 5 s in V4 for the imagery condition (Fig. S7).

### Imagery decoding as a function of reported vividness

Next, we investigated the effect of subjective imagery vividness on decoding accuracy for imagery. We divided the trials into low- and high-vividness (mean split, see Materials and Methods for details). As expected, the decoding accuracy for imagery content was higher in high-vividness trials, but surprisingly, the strongest differences were observed before the onset of imagery (Fig. S8A). The generalization analysis showed the same trend. We found above chance decoding only in high vividness trials (Fig. S8B), suggesting that in more vivid imagery trials, shared representations between perception and imagery would emerge more readily before volition. This result suggests that the subjective strength of future imagery is associated with better predictive power in visual areas.

### Decoding future imagery vividness from pre-imagery responses

**Figure 6:**
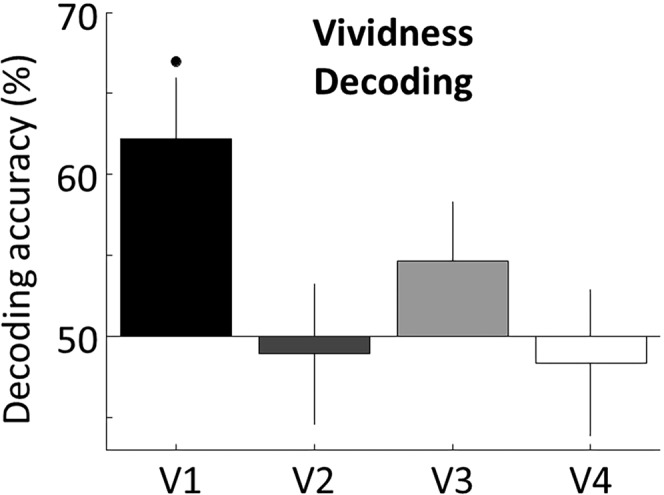
Pre-imagery activation patterns in the primary visual cortex (V1) predict the strength of subsequent visual thoughts. We used pre-imagery data (from −10 to 0 s from the voluntary imagery onset) to decode subsequent imagery vividness (high vs low, see text for details). Information in V1 from before the imagery decision predicted how vivid the future imagery will be. Error bars represent SEM across participants. Full point represents above chance decoding (p = 0.0035, one-sample t-test against chance: 50%).

Finally, we reasoned that if prior, pre-imagery sensory representations in early visual cortex do indeed dictate the strength of subsequent visual imagery, then the pre-imagery data should predict the reported vividness from the subsequent imagery period. Accordingly, we tested exactly this, we attempted to decode the subjective strength of imagery (i.e. vividness) by using only the fMRI data from before the imagery period (Fig. 6). Decoding accuracy was significantly above chance in V1 (62.2%, p = 0.0035, one-sample, one-tailed t-test against chance: 50%), but not in other visual ROIs (p > 0.05, Fig. 6), indicating that information contained in V1 predicted future subjective imagery strength. This result shows that the predictive information in primary visual cortex not only has an influence on the contents of future imagery, but also impacts the subjective quality of the future visual thought.

## Discussion

We found that neural activation patterns were predictive of the contents of voluntary visual imagery as far as −11 seconds before the choice of what to imagine. These results suggest that the contents of future visual imagery can be biased by current or prior neural representations.

While previous interpretations have assigned predictive signals an unconscious origin^7,9,32,33^, we remain agnostic as to whether predictive signals were accompanied by awareness or not. We acknowledge the inherent limitations of most paradigms at capturing the state of awareness of the participants before their decision (see for example^17^). We have nonetheless gone to great lengths to overcome these limitations by developing a behavioral test aimed at probing the accuracy of imagery onset reports (Fig. 3). While this independent experiment suggested that participants were not imagining the gratings before the reported onset, the experiment does not completely exclude the possibility that participants engaged in imagery before the reported onset while in the scanner.

Our results show predictive patterns in occipital, frontal and subcortical areas (Fig. 2). While previous results highlight the role of frontal areas carrying information about subsequent decisions^7,9,10^; to the best of our knowledge, predictive signals in visual and subcortical areas have not been reported. Interestingly, recent results have shown that brainstem centers can be a source of variability in the form of biases in perceptual decisions due to arousal mechanisms^34^. A similar mechanism could support the involvement of subcortical regions in the bias of future visual imagery.

### Predictive signals in visual areas have perceptual properties

Are the predictive signals low-level visual representations or more abstract signals? Our results suggest that the predictive signals share properties with perceptual representations. Two pieces of evidence support this interpretation. First, predictive signals were found in visual areas V1, V3 and V4, which are devoted to visual processing, thus suggesting that predictive information has perceptual properties (Fig. 4). Secondly, and more conclusively, using brain signals elicited by unattended perceptual gratings, we were able to classify the contents of imagery before the decision (Fig. 5). The result of this generalization decoding analysis shows that predictive information in V1 shares similarities with perception, thus suggesting that these signals correspond, at least partly, to visual representations.

As for the specific features coded by the predictive sensory-like representations, it is unclear whether they correspond to orientation, color or to the conjunction of both. This question can however be answered by future experiments on perception-imagery generalization cross-decoding by using perceptual stimuli in the form of greyscale oriented gratings and solid color patches, while imagining the same colored oriented gratings as in the current study. Such a design would be able to distinguish the specific feature content of these representations: differences in decoding accuracy between color patch-imagery and achromatic gratings-imagery generalization should shed light on which features are coded by the pre-volitional signals.

### Timing of the predictive signals and possible confounds

The finding of predictive signals up to 11 seconds before the decision can seem surprisingly early. However, early predictive signals have been detected using similar techniques in previous studies on motor decisions (up to 10 and 7 seconds before decision^7,9^) and also on abstract decisions (up to 4 seconds^10^). Crucially, questions have been raised about whether the decoding of such signals can correspond to neural activity elicited by the preceding trial. The N-1 trial shifting or “spillover” analysis performed in our study (Figs S5 and S7), is an accepted way to control for this issue^19,35^. The spillover analysis tests the hypothesis that if there is a temporal carry-over of information from one trial to the next, predictive signals should be best accounted by shifting the label of the current trial to the trial before (see Materials and Method for details). Results of the spillover control analysis showed that the predictive signals in our study are not explained by the previous trial, thus dismissing spillover effects as an explanation of our data (Figs S5 and S7).

Another relevant issue is that the sequential dependencies might have an impact on the classifier itself. In other words, any deviation from randomness in the choice across trials (captured for example by the entropy value or the probability of switch) could be potentially exploited by the classifier. Previous studies have shown that classifiers trained only on behavioral responses can perform as well as or better than classification on neural responses^20,36^. While sequential dependencies have been argued to be negligible on previous experiments^21^, this issue is difficult to dismiss without independent experiments. While, in our experiment, the probabilities of choosing vertical or horizontal were very similar (50.44% and 49.56%, Shannon entropy = 0.997) the probability of switching gratings from one trial to the next deviated from chance (58.59%). Therefore, by taking the results from the imagery decoding alone, we cannot rule out that sequential dependencies could have influenced the classification, as the classifier would have reached 58.59% decoding accuracy just by predicting that the decision on next trial would be switched from the previous one. Crucially, our independent perception-imagery generalization decoding analysis does not suffer from sequential dependencies issues as classifiers were trained exclusively on perception trials (presented in a 15s-on/15s-off block design) and tested on imagery trials. The perception-imagery generalization decoding confirmed predictive signals before the decision from (Figs 5 and S8B), thus indicating that our results are not explained by sequential dependencies in the participants’ choices.

### Predictive information in the primary visual cortex (V1) impacts the subjective strength of future imagery

Interestingly, information contained in the primary visual cortex (V1) predicted the subjective strength of visual imagery (Fig. 6). This suggests that the phenomenology of future mental images is supported by patterns of activations in the primary visual cortex that are present before the onset of voluntary imagery. This result again, links information contained in visual areas with the subjective properties of future voluntary imagery.

### Choice prediction can be explained by decisions relying on spontaneously generated representations

In previous experiments applying MVPA to study decision processes, predictive information about choices has been interpreted as evidence for nonconscious decision making^7–9^. Thus, it could be possible to interpret our results as the imagery decision being made (at least partly) non-consciously, supporting the idea that subjective sensation of making the decision emerges after the decision is already made^7,9,32,33^.

An alternative hypothesis is that these results reflect decisional mechanisms that rely on spontaneously generated visual representations present before the decision. Since the goal of the task was to randomly choose and imagine a grating as vividly as possible, one strategy might be to choose the pattern that is spontaneously more strongly represented. In other words, spontaneous grating representations might stochastically fluctuate in strength while remaining weak compared to voluntary imagery. Thus, prior to the decision, one grating representation might dominate, hence being more prone to decisional thought-selection. An analogous interpretation has been advanced to explain the buildup of neural activity prior to self-initiated movements, aka readiness potential^37^.

Interestingly, it has been recently shown that self-initiated movements can be aborted even after the onset of predictive neural signals^38^, suggesting that the decision can be somewhat dissociated from predictive neural signals. Therefore, our results can be explained by a conscious choice that relies on weak neural representations during the decision production; perhaps analogous to blindsight^39^, subliminal priming studies^40,41^ or nonconscious decisional accumulation^42^. Such a mechanism is intriguing in light of theories of mental imagery and thought generation that propose involuntary thought intrusion as both an everyday event, and, in extreme cases, a component of mental disorders like PTSD^43,44^.

In summary, we think that the best way to explain our results is not in terms of unconscious decision processes (as it has been advanced previously in the literature), but rather by a process in which a decision (which could be conscious) is informed by weak sensory representations.

### Concluding remarks and future directions

Our current study can be seen as the first to capture the possible origins and contents of involuntary thoughts and how they progress into or bias subsequent voluntary imagery. This is compatible with the finding that the most prominent differences between low and high vividness trials are seen for the pre-imagery period in visual areas, especially the primary visual cortex, which can be interpreted as when one of the patterns is more strongly represented it will induce a more vivid subsequent volitional mental image. This is in line with reports showing that imagery vividness depends on the relative overlap of the patterns of activation elicited by visual perception and imagery^45^. Our results expand that finding by showing that the vividness of future visual thoughts is predicted by information stored in the primary visual cortex.

It is up to future research to reveal whether representations biasing subsequent voluntary imagery are genuinely non-conscious or not. This will not only shed light on age-old questions of volition, but also provide a clear mechanism for pathological intrusive thoughts common across multiple mental disorders.

## Material and Methods

### Participants

Experimental procedures were approved by the University of New South Wales Human Research Ethics Committee (HREC#: HC12030). All methods in this study were performed in accordance with the guidelines and regulations from the Australian National Statement on Ethical Conduct in Human Research (https://www.nhmrc.gov.au/guidelines-publications/e72). All participants gave informed consent to participate in the experiment. For the fMRI experiment, we tested 14 participants (9 females, aged 29.1 ± 1.1 years old, mean ± SEM). We selected the sample size based on both estimations of effect sizes and the number of participants used in previous studies employing decoding to track brain signals predictive of subsequent decisions^7–9^. Previous works tested from 8 to 14 participants, we thus used the participant’s number upper bound in order to maximize the reliability of the results. We performed power analyses to corroborate that this number of participants was adequate to achieve a power of at least 0.8. Based on effect size estimations using G*Power 3^46^. Soon at al. study on the pre volitional determinants of decision making^9^ tested 14 participants achieving a power of 0.812 in the time resolved decoding analysis while Bannert and Bartels study on perception-imagery cross-decoding generalization tested 8^30^. Post hoc effect size analysis revealed that they would have needed to test 12 participants to achieve a power of 0.8. For the behavioral free decision and cued imagery priming task, we invited all the previous 14 participants to be part in this psychophysics experiment. Only 8 participants (4 females, aged 29.3 ± 0.5 years old), were able to come back to complete this new experiment.

### fMRI free decision visual imagery task

We instructed participants to choose between two predefined gratings (horizontal green/vertical red or vertical green/horizontal red, counterbalanced across participants), which were previously familiar to the participants through prior training sessions. We asked the participants to refrain from following preconceived decision schemes. In the scanner, participants were provided with two dual-button boxes, one held in each hand. Each trial started with a prompt reading: “take your time to choose – press right button” for 2 seconds (Fig. 1). After this, a screen containing a fixation point was shown while the decision as to what to think of was made. This period is referred as “pre-imagery time” and was limited to 20 seconds. Participants were instructed to settle their mind before deciding. Participants pressed a button with the right hand as soon as they decided which grating to imagine. Participants reported that in some trials they felt in control of their decision, whereas in other trials one of the gratings just “popped-out” in their mind. Importantly, participants were instructed to press the button as soon as possible when they reached the decision or a grating appeared in their mind. After pressing the button, the fixation point became brighter for 100 ms indicating the participants that the imagery onset time was recorded. During the imagery period (10 seconds), participants were instructed to imagine the chosen pattern as vividly as possible, trying, if possible, to project it onto the screen. At the end of the imagery period, a question appeared on the screen: “what did you imagine? – Left for vertical green/red – Right for horizontal red/green” (depending on the pre-assigned patterns for the participant). After giving the answer, a second question appeared: “how vivid was it? – 1 (low) to 4 (high)” to which participants answered using 4 different buttons. After each trial, there was a blank interval of 10 seconds where we instructed the participants to just relax and try not to think about the gratings nor any subsequent decisions. Systematic post-experiment interviews revealed that some participants (n = 4) could not help thinking about gratings in some trials during the inter trial interval. They reported different strategies to avoid these thoughts such as ignoring them, replacing them for another image/thought, or choosing the other grating when the decision came. The remaining participants (n = 10) reported not having any thoughts or mental images about gratings during the rest period. We tested if the effects we found could be explained by the former group of participants who could not refrain from thinking about gratings. We thus performed the analysis using only data from the participants who did not think/imagine gratings outside the imagery period (n = 10). Fig. S10 shows the results of this control. Results are comparable to those shown in Fig. 2, thus ruling out the possibility that that the effects we report were driven by the 4 participants who had thoughts about gratings in the rest period. We delivered the task in runs of 5 minutes during which the participants completed as many trials as possible. Participants chose to imagine horizontal and vertical gratings with a similar probability (50.44% versus 49.56% for vertical and horizontal gratings respectively, mean Shannon entropy = 0.997 ± 0.001 SEM) and showed an average probability of switching gratings from one trial to the next of 58.59% ±2.81 SEM. Participants completed in average 7.07 runs each, with each run containing an average of 9.2 trials.

### Behavioral imagery onset reliability experiment

Since the self-report of the onset of decisions has been criticized due to its unreliability and unknown variance^17^, we developed a novel independent psychophysics experiment to test its reliability. We objectively measured imagery strength as a function of time for a subset of the participants from the fMRI experiment. Importantly, the results of this experiment revealed that the reported onsets of decisions are indeed reliable relative to the temporal resolution of the fMRI (Fig. 3).

We employed two conditions: free decision (freely chosen imagined stimulus and imagery onset) and cued (i.e., imposed imagined stimuli and imagery onset), see Fig. 3A for a schematic of the paradigm. We used binocular rivalry priming as a means to objectively measure sensory imagery strength^18,47,48^. When imagining one of the two competing rivalry stimuli prior to a binocular rivalry presentation, rivalry perception is biased towards the imagined stimulus, with greater levels of priming as the imagery time increases^18^; see^18,28^ for discussion of why this is an objective measure of imagery strength and not visual attention, binocular rivalry control or response bias. We asked participants to imagine one of the rivalry gratings for different durations and then measured rivalry priming as a function of the different imagery durations (Fig. 3B). We reasoned that if participants reported the onset of imagery a few seconds after they actually started imagining, this would be detected as an increase in priming compared to the condition where the onset of imagery is controlled by the experimenter. Thus, in the free decision condition, participants had to freely choose to imagine one of the two predefined gratings (horizontal green/vertical red or vertical green/horizontal red, counterbalanced across participants). In the cued condition, participants were presented with a cue indicating which grating to imagine, thus imposing the onset of imagery as well as which grating needed to be imagined. Each trial started with the instruction “press spacebar to start the trial” (Fig. 3A). Then, either the instruction “CHOOSE” or a cue indicating which grating to imagine (i.e., “horizontal red”) was presented for 1 second. In the free decision condition, the imagery time started after the participant chose the grating to imagine, which they indicated by pressing a key on the computer keyboard (Fig. 3A). For the cued imagery condition, the imagery time started right after the cue was gone (i.e., no decision time). We tested 3 imagery times (3.33, 6.67 and 10 seconds). After the imagery time, a high pitch sound was delivered (200 ms) and both gratings were presented through red/green stereo glasses at fixation for 700 ms. Then, participants had to report which grating was dominant (i.e., horizontal red, vertical green or mixed if no grating was dominant), by pressing different keys. After this, they had to answer which grating they imagined (for both free decision and cued trials). Participants then rated their imagery vividness from 1 (low) to 4 (high) by pressing one of the 4 buttons in their response boxes. Free decision and cued trials as well as imagery times were pseudo-randomized within a block of 30 trials. We added catch trials (20%) in which the gratings were physically fused and equally dominant to control the reliability of self-report^18,49^. We tested 120 trials for each free decision and cued imagery conditions (40 trials per time point), plus 48 catch trials evenly divided among time points.

Raw priming values were calculated as the number of congruent dominant gratings in binocular rivalry (e.g., imagined vertical led to vertical dominant in binocular rivalry) divided by the total number of trials excluding mixed dominance binocular (piecemeal), for each time point and condition independently. Raw vividness values were calculated as the average per time point and condition excluding mixed perception trials. Priming and vividness were normalized as z-score within participants and across time-points and conditions to account for baseline differences across participants, but otherwise conserving relative differences amongst conditions and time-points. Rivalry dominance self-report reliability was verified with fake rivalry catch trials, where gratings were physically fused and equally dominant, which were reported as mixed above chance level (83.8%, p = 0.002, one-sample t-test against baseline). Priming and vividness z-scores were subjected to a one-way ANOVA to detect main the effects of conditions. We also performed post-hoc two-sample t-tests to verify that priming and vividness scores differed significantly between time points (Fig. 3).

We tested this independent behavioral experiment on 8 participants from the fMRI experiment (all 14 original participants were invited but only 8 were able to come back), who had extensive experience as subjects in psychophysics experiments. We further sought to test if these results would generalize to completely inexperienced participants who did not participate in the fMRI experiment (N = 10). We did not, however, find a significant increase of priming or vividness as a function of time as for results on Fig. S11, suggesting that this is a highly demanding task and experience in psychophysics might be important to perform the task properly (i.e., being able to hold the mental image for the duration of the imagery time).

### Functional and structural MRI parameters

Scans were performed at the Neuroscience Research Australia (NeuRA) facility, Sydney, Australia, in a Philips 3T Achieva TX MRI scanner using a 32-channel head coil. Structural images were acquired using turbo field echo (TFE) sequence consisting in 256 T1-weighted sagittal slices covering the whole brain (flip angle 8 deg, matrix size = 256 × 256, voxel size = 1 mm isotropic). Functional T2*-weighted images were acquired using echo planar imaging (EPI) sequence, with 31 slices (flip angle = 90 deg, matrix size = 240 × 240, voxel size = 3 mm isotropic, TR = 2000ms, TE = 40 ms).

### fMRI perception condition

We presented counter-phase flickering gratings at 4.167 Hz (70% contrast, ~0.5 degrees of visual angle per cycle). They were presented at their respective predefined colors and orientations (horizontal green/vertical red or vertical green/horizontal red). The gratings were convolved with a Gaussian-like 2D kernel to obtain smooth-edged circular gratings. Gratings were presented inside a rectangle (the same that was used in the imagery task, Fig. 1) and a fixation point was drawn at the center (as for the imagery task). Within a run of 3 minutes, we presented the flickering patterns in a block manner, interleaved with fixation periods (15 seconds each). Importantly, an attention task was performed consisting of detecting a change in fixation point brightness (+70% for 200 ms). Fixation changes were allocated randomly during a run, from 1 to 4 instances. Participants were instructed to press any of the 4 buttons as soon as they detected the changes. Participants showed high performance in the detection task (d-prime = 3.33 ± 0.13 SEM).

### Functional mapping of retinotopic visual areas

To functionally determine the boundaries of visual areas from V1 to V4 independently for each participant, we used the phase-encoding method^50,51^. Double wedges containing dynamic colored patterns cycled through 10 rotations in 10 min (retinotopic stimulation frequency = 0.033 Hz). To ensure deployment of attention to the stimulus during the mapping, participants performed a detection task: pressing a button upon seeing a gray dot anywhere on the wedges.

### Experimental procedures

We performed the 3 experiments in a single scanning session lasting about 1.5 h. Stimuli were delivered using an 18” MRI-compatible LCD screen (Philips ERD-2, 60 Hz refresh rate) located at the end of the bore. All stimuli were delivered and responses gathered employing the Psychtoolbox 3^52,53^ for MATLAB (The MathWorks Inc., Natick, MA, USA) using in-house scripts. Participants’ heads were restrained using foam pads and adhesive tape. Each session followed the same structure: first the structural scanning followed by the retinotopic mapping. Then the perception task was alternated with the imagery task until completing 3 runs of the perception task. Then the imagery task was repeated until completing 7 or 8 (depending on the participant) runs in total. Pauses were assigned in between the runs. The 4 first volumes of each functional runs were discarded to account for the equilibrium magnetization time and each functional run started with 10 seconds of fixation.

### Phase-encoded retinotopic mapping analysis

Functional MRI retinotopic mapping data were analyzed using the Fast-Fourier Transform (FFT) in MATLAB. The FFT was applied voxel-wise across time points. The complex output of the FFT contained both the amplitude and phase information of sinusoidal components of the BOLD signal. Phase information at the frequency of stimulation (0.033 Hz) was then extracted, using its amplitude as threshold (≥2 SNR) and overlaid them on each participant’s cortical surface reconstruction obtained using Freesurfer^54,55^. We manually delineated boundaries between retinotopic areas on the flattened surface around the occipital pole by identifying voxels showing phase reversals in the polar angle map, representing the horizontal and vertical visual meridians. In all participants, we clearly defined five distinct visual areas: V1, V2, V3d, V3v and V4; throughout this paper, we merge V3d and V3v and label them as V3. All four retinotopic labels were then defined as the intersection with the perceptual blocks (grating > fixation, p < 0.001, FDR corrected) thus restricting the ROI to the foveal representation of each visual area.

### Functional MRI signal processing

All data were analyzed using SPM12 (Statistical Parametric Mapping; Wellcome Trust Centre for Neuroimaging, London, UK). We realigned functional images to the first functional volume and high-pass filtered (128 seconds) to remove low-frequency drifts in the signal, with no additional spatial smoothing. To estimate the hemodynamic response function (HRF), we generated regressors for each grating (horizontal green/vertical red or vertical green/horizontal red) for each run and experiment (perception and imagery) independently. We used finite-impulse response (FIR) as the basis function. This basis function makes no assumptions about the shape of the HRF which is important for the analysis of the free decision imagery data^9^. We employed a 14th order FIR basis function encompassing 28 seconds from −13 to +13 seconds from the imagery onset, thus obtaining 14 bins representing each TR. For the perception condition, we employed a 1^st^ order FIR basis function from the onset of each perceptual block to its end (15 seconds). We also employed 1^st^ order FIR basis functions for the sanity check imagery decoding (from 0 to 10 s, Fig. S2) and the before-after decision perception-imagery generalization (−10 to 0 and 0 to 10 from imagery decision, Fig. 5). For the vividness analysis, we split the trials into low-vividness (ratings 1 and 2) and high-vividness (ratings 3 and 4), we then obtained the regressors for both gratings as explained above.

### Multi-voxel pattern analysis (MVPA)

We used a well-established decoding approach to extract information related to each grating contained in the pattern of activation across voxels of a given participant (in their “native” anatomical space) using the decoding toolbox (TDT)^56^. Using a leave-one-run out cross-validation scheme, we trained a L2-norm regularized linear supporting vector machine (SVM, as implemented in LIBSVM) on beta values using all but one run and then tested on the remaining one. No additional scaling (normalization) was performed on the data as beta values represent a scaled version of the data relative to the run mean. Training and testing was repeated until all runs were used as test and then averaged the results across validations (7 or 8-fold, depending on the participant). We performed leave-one-run out cross validation for every temporal bin independently.

We also employed cross-classification to generalize information between the perception and the imagery tasks in the “perception-imagery generalization”.

For the perception-imagery cross-classification, we trained on the ensemble of the perception runs and tested on the ensemble of the imagery runs. In each perception run, green and red gratings were shown pseudorandomly in 6 blocks of 15 s each. Perceptual blocks (15 s) were convolved with a 1^st^ order FIR filter, yielding regressors for red and green perceptual gratings, as explained in the previous section. Imagery trials were pre-processed exactly as in the imagery decoding, yielding time-resolved (2 s) or block (10 s) regressors (see previous section for details). Thus, classifiers trained on the perceptual runs (e.g., perceptual vertical-green vs perceptual horizontal-red) were tested on the imagery data (e.g., imagined vertical-green vs imagined horizontal-red). Accuracy was calculated as in the imagery decoding (e.g., percentage of vertical-green vs horizontal-red decoding accuracy), except for that the training-testing procedure was performed only once (i.e., all perceptual data was used to train and all imagery data was used to test the classifiers), since it is not necessary to use cross-validation in such cross-classification schemes as the training and testing data are different and independent (as opposed to the imagery decoding condition where a fraction of the data was used for training and another for testing).

We employed 2 different decoding approaches: searchlight and region-of-interest (ROI). We used a spherical searchlight of 3 voxels of radius and obtained volumes in which a value of decoding accuracy was assigned to each voxel. We normalized the decoding accuracy volumes into the MNI space and applied a spatial smoothing of 8 mm FWHM, which has been found to be optimal in order to account for anatomical idiosyncrasies across participants^57^. We then performed a one-tail one-sample t-test against 50% (chance level) across participants for every voxel. We corrected for multiple comparisons using cluster-extent based thresholding employing Gaussian Random Field theory^58,59^, as implemented in FSL^60^. We used a primary threshold of p < 0.001 at the voxel level, as recommended in previous studies^61^, and a cluster level threshold of p < 0.05 in every time point volume independently. Importantly, these thresholds have been shown to be valid within the nominal false positive ratios^62^.

ROI decoding was used to test information content in visual areas specifically. We defined the boundaries of visual areas from V1 to V4 which volumes were used as ROI. Note that because visual ROI were defined on the cortical surface (see phase-encoded retinotopic analysis for details), only gray-matter containing voxels were considered, as opposed to the searchlight approach which also considers non-gray matter containing voxels, potentially explaining differences on sensitivity between these approaches.

We tested if there was a difference in the average BOLD response between stimuli (i.e., univariate difference). We did not find any significant differences (p > 0.05, uncorrected) in the average BOLD response (Fig. S9), thus ruling-out the possibility that the results would be explained by differences in the average level of activity across conditions.

### Permutation test

In order to validate the use of standard parametric statistics, we performed a permutation test and thus empirically determined the distribution of decoding accuracies under the null hypothesis^63^. Previous reports have highlighted the possibility of obtaining skewed decoding distributions, which would invalidate the use of standard parametric statistical tests^29^. We thus randomly shuffled the labels (i.e., horizontal red/vertical green) among trials and within blocks (i.e., number of red/green imagined trials was conserved within a run but trial labels were shuffled) for each participant and condition (imagery and generalization) to generate empirical data under the null hypothesis. After reshuffling the labels, we generated regressors for each stimulus and performed decoding following the same procedure described in the previous paragraph. We repeated this procedure 1000 times and obtained the empirical distribution under the null hypothesis. At each iteration, the second level analysis (across participants) consisted of averaging the results across participants (exactly as performed on the original data), from which we obtained confidence intervals for each decoding time point and area (Figs S4 and S6) using the percentile method^63^. Our results show that the decoding null hypothesis followed a normal distribution (Table S2) and importantly, significant results using permutation test confidence intervals were comparable to the results using standard parametric tests (compare significant points on Figs 2 and 3 with Figs S4 and S6). This analysis thus validates the use of standard statistical tests to test significance on our dataset.

### Across time-points family-wise error rate (FWER) control

We estimated the probability of obtaining an n number of significantly above-chance decoding time points (p < 0.05, one tailed t-test) under the null hypothesis. To do this, we employed the data from the null distribution obtained with the permutation test (randomly shuffled labels, 1000 iterations; see previous paragraph for details). Fig. S3 shows the result of such analysis. Insets show the family-wise error rate for the empirically observed number above-chance decoding time points for each area.

### Spillover effect (N-1) decoding control

We conducted a control analysis to directly test whether the searchlight results could be explained by any spill over from the previous trial, as performed in a previous study (Soon *et al*.^19^). To do this, we shifted the labels by one trial (N-1). Briefly, the rationale behind this control is the following: if there was spill over from the previous trial, this analysis should show higher decoding accuracy in the pre-imagery period as effects from the previous trial would spillover over the next trial (for a comprehensive explanation of the rationale please refer to Soon *et al*.^19^). All the decoding details were otherwise identical to what is described in the section “Multi-voxel pattern analysis (MVPA)” except for that the first trial of each run was not considered as there was no N-1 trial in that case. Analogously, for the perception-imagery generalization, training was performed on perception data and tested on imagery trials labeled as N-1.

## Supplementary information

Supplementary Figures and Tables

## Data Availability

The datasets generated during and/or analysed during the current study are available from the corresponding author on reasonable request.
