# Supplementary material for: Decoding the contents and strength of imagery before volitional engagement
Source: Sci Rep. 2019 Mar 5;9:3504. doi: 10.1038/s41598-019-39813-y (PMC6401098; doi:10.1038/s41598-019-39813-y)
Supplement: Supplementary file 1 — Supplementary Figures and Tables [file 41598_2019_39813_MOESM1_ESM.pdf]

Decoding the contents and strength of imagery before volitional engagement

Roger Koenig-Robert and Joel Pearson

Supplementary Figures and Tables

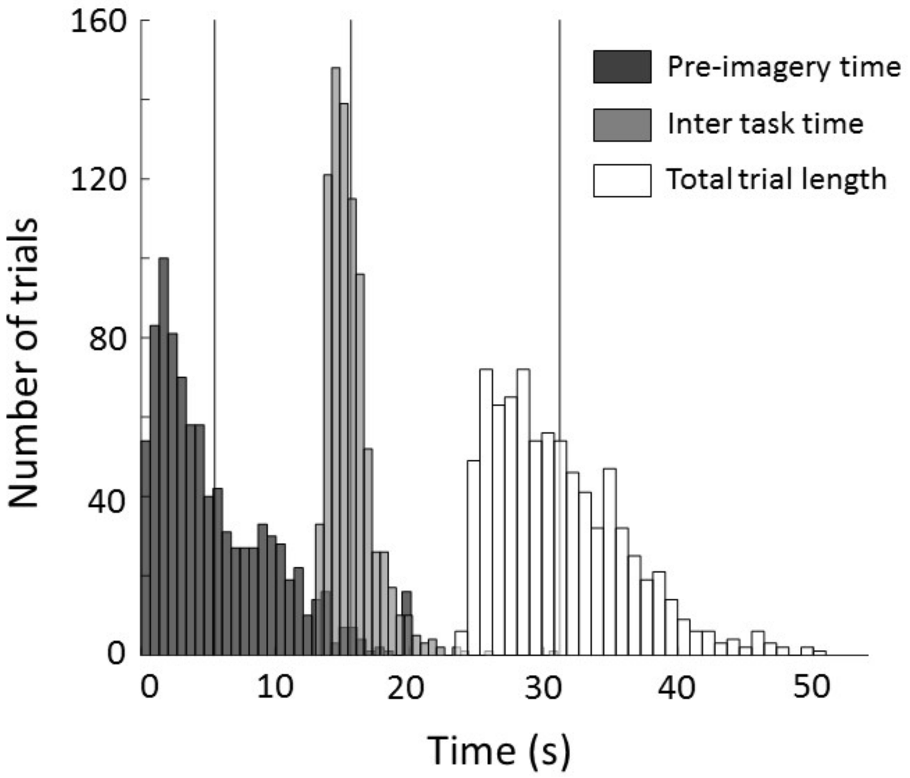

Figure S1. **Distributions of the periods composing the free-decision task in the fMRI experiment.** Pre-imagery time corresponds to the interval from the end of the trial-start instructions ("take your time to choose / press right button", presented for 2s) to the button press, indicating the start of the imagery period. The Inter task period corresponds to the lapse between the end of the imagery period from one trial to the start of the pre-imagery period in the next trial. Total trial length was defined from onset to onset of the trial instructions in consecutive trials. Vertical lines represent the mean of every period. Average times for pre-imagery, inter task and total trial length were 5.48s, 15.62s and 31.18s, respectively. Note that these time intervals left enough time between trials to avoid activity spill over.

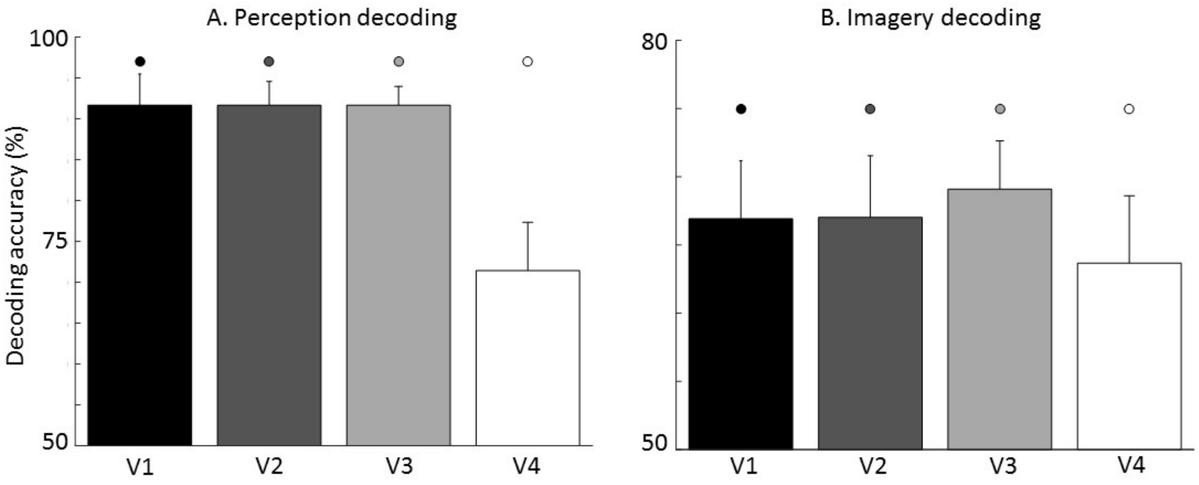

Figure S2. **Sanity check of the decoding of perception and imagery contents.** We validated our classification approach by decoding perception (A) and imagery (B) on visual ROI. A leave-one-run cross validation scheme was used to train and test linear classifiers (SVM). Perception fMRI data was extracted from 10s of perception blocks. Imagery fMRI data were extracted from the 10s imagery time in the free decision task. Perception (91.7, 91.7, 91.7 and 71.4%; from V1 to V4) and imagery (66.9, 67, 69.1 and 63.7%) decoding accuracy was comparable to previous reports [1–3], thus validating our classification approach. Error bars represent SEM across participants. Dots represent above chance decoding (chance level=50%, p<0.01).

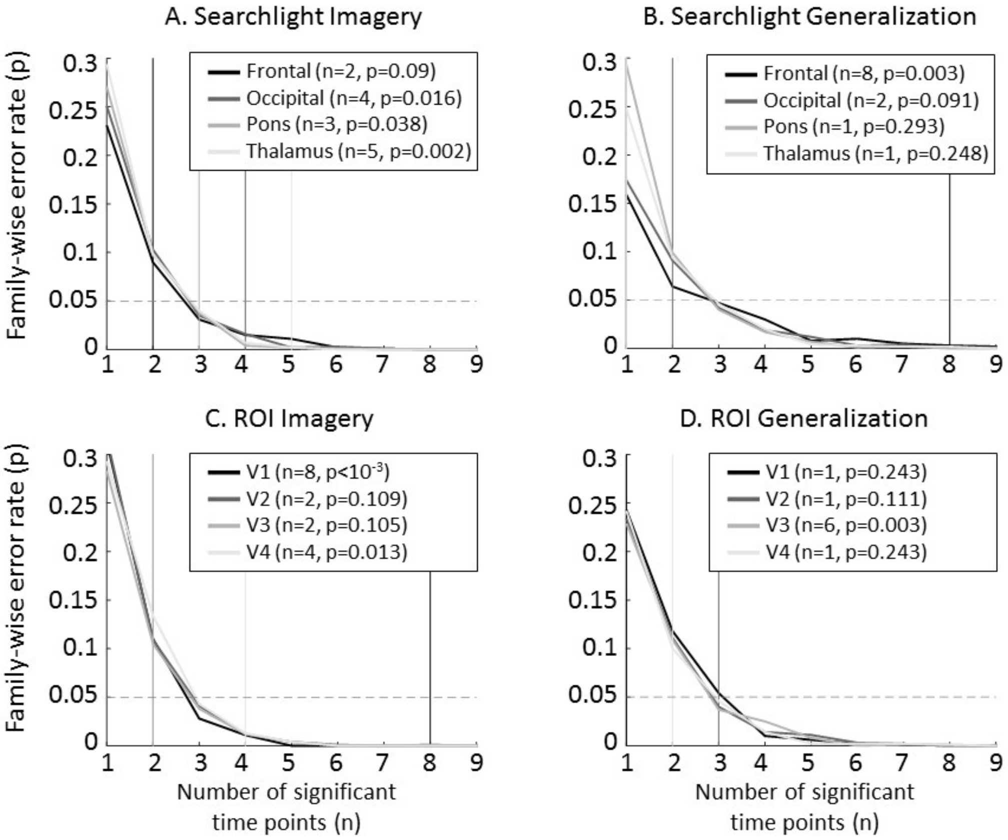

Figure S3. **Assessment of the family-wise error rate across time points.** We estimated the probability of obtaining different number (n, from 1 to 9) of significantly above chance decoding across time points ($p<0.05$, one tailed t-test) under the null hypothesis using the null distribution from the permutation test (1000 iterations). Insets show the family-wise error rate for the empirically observed number above-chance decoding time points for each area.

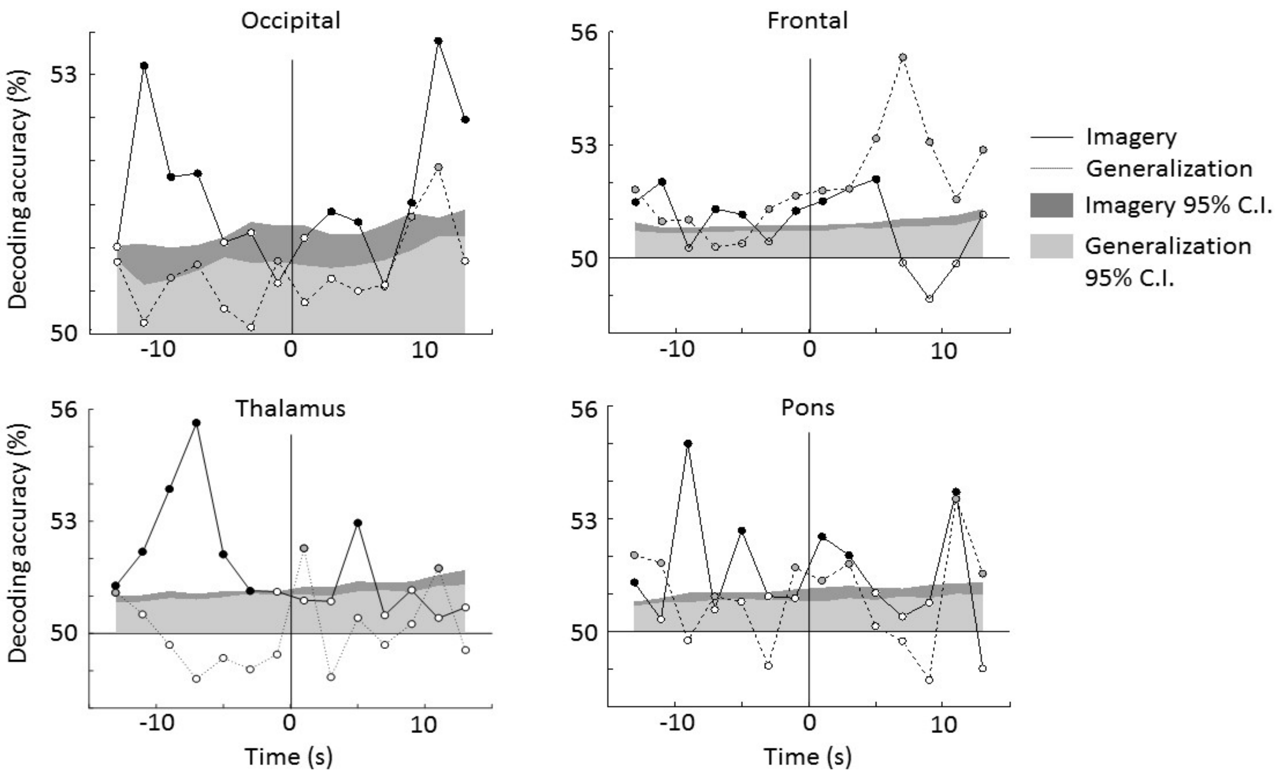

Figure S4. **Searchlight confidence intervals (C.I.) from permutation test.** We validated the statistical significance of the searchlight decoding accuracy by empirically determining the distribution of the null hypothesis. We thus performed a permutation test (1000 iterations) for each participant and decoding method independently by randomizing the labels (horizontal/vertical, red/green) from trials prior the construction of regressors (see text for details). Confidence intervals (95%, right-tailed) for imagery and generalization are shown in dark and light gray, respectively. Significant above-chance decoding time-points ($p<0.05$, right-tailed permutation test) are depicted as solid circles. Results are comparable to those shown in Figure 2 in which parametric tests were used (t-test, one-sample, right-tailed), thus validating the use of parametric statistical tests.

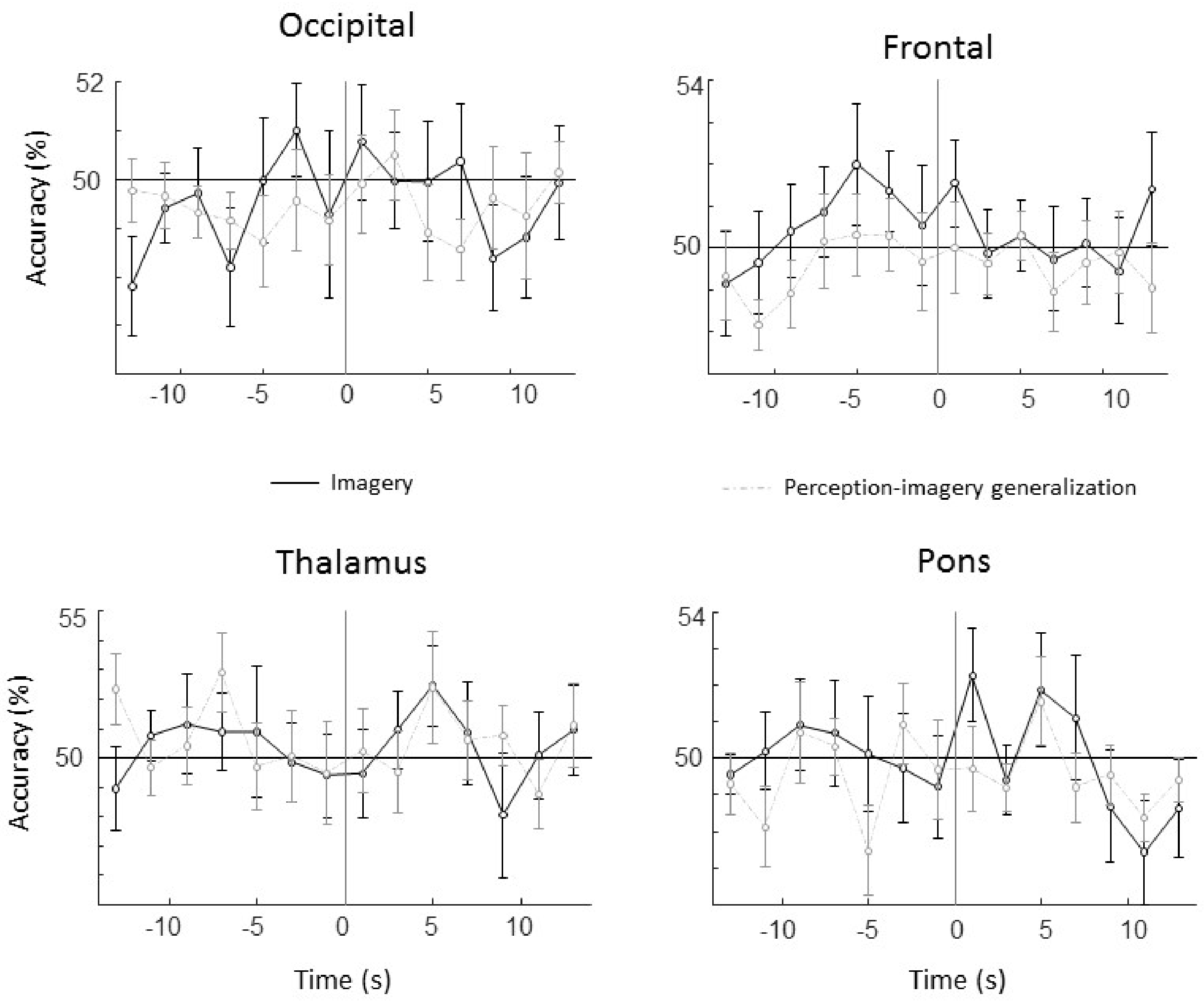

Figure S5. **Searchlight spillover effect control.** We conducted a control analysis to test whether our results could be explained by activity from the previous trial (spillover effect) for imagery and perception-imagery generalization (black and gray curves, respectively). We thus trained and tested our classifiers on data with shifted labels (N-1). If there was a spill over from previous trial, this analysis should show higher decoding accuracy in the pre-imagery period compared to the original analysis (for details on the rationale see Materials and Methods section and Soon, Allefeld, Bogler, Heinzle, & Haynes, 2014). We found no significant above chance classification for any of the regions ($p>0.05$, one-tailed t-test), thus ruling out the possibility that these results are explained by any spill over. Error bars represent $\pm$SEM across participants.

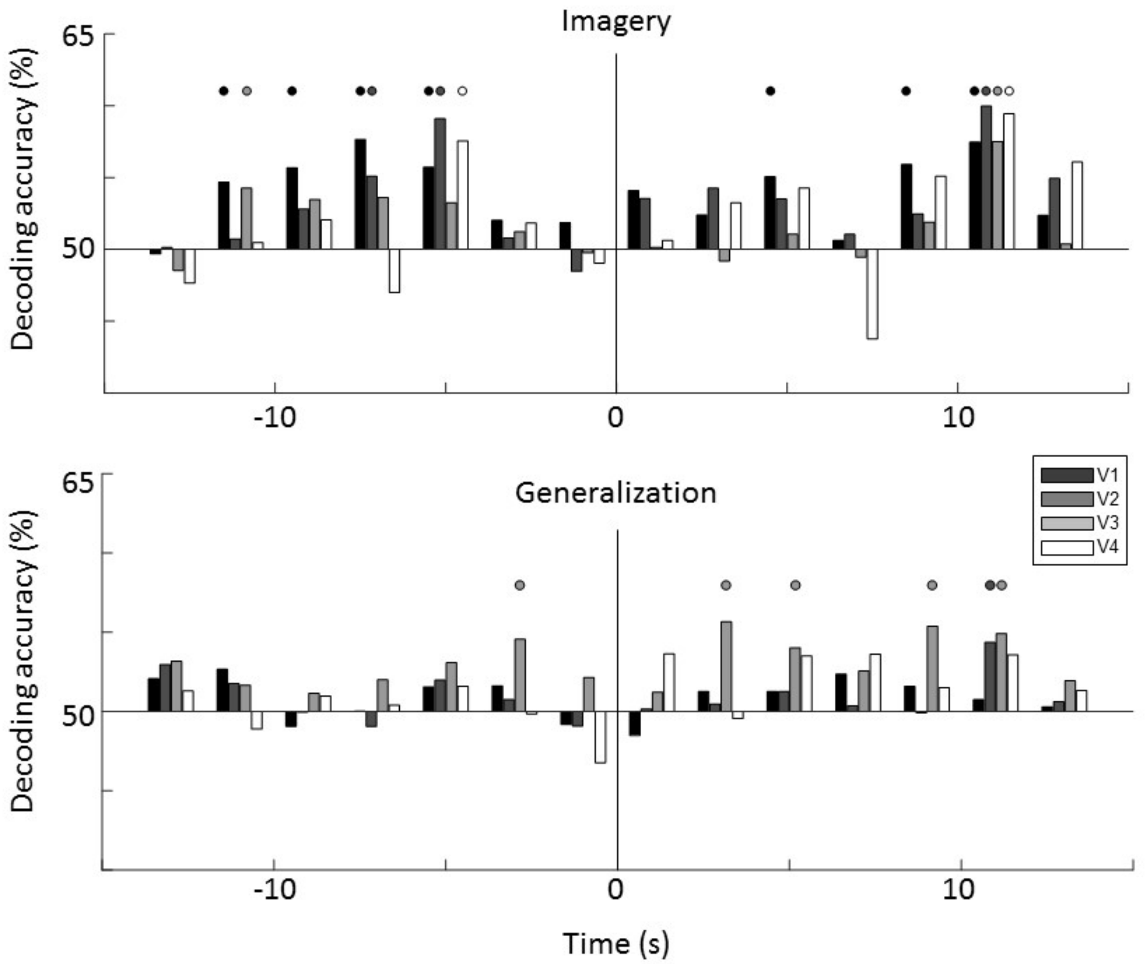

Figure S6. **ROI results from permutation test.** We validated the statistical significance of the ROI decoding accuracy by empirically determining the distribution of the null hypothesis. We thus conducted a permutation test (1000 iterations) for each participant, decoding method and time-point independently by randomizing the labels (horizontal/vertical, red/green) from trials prior the construction of regressors (see text for details). Significant above-chance decoding time-points ($p<0.05$, right-tailed permutation test) are depicted as solid circles. Results are comparable to those shown in Figure 4 in which parametric tests were used (t-test, one-sample, right-tailed), thus validating the use of parametric statistical tests.

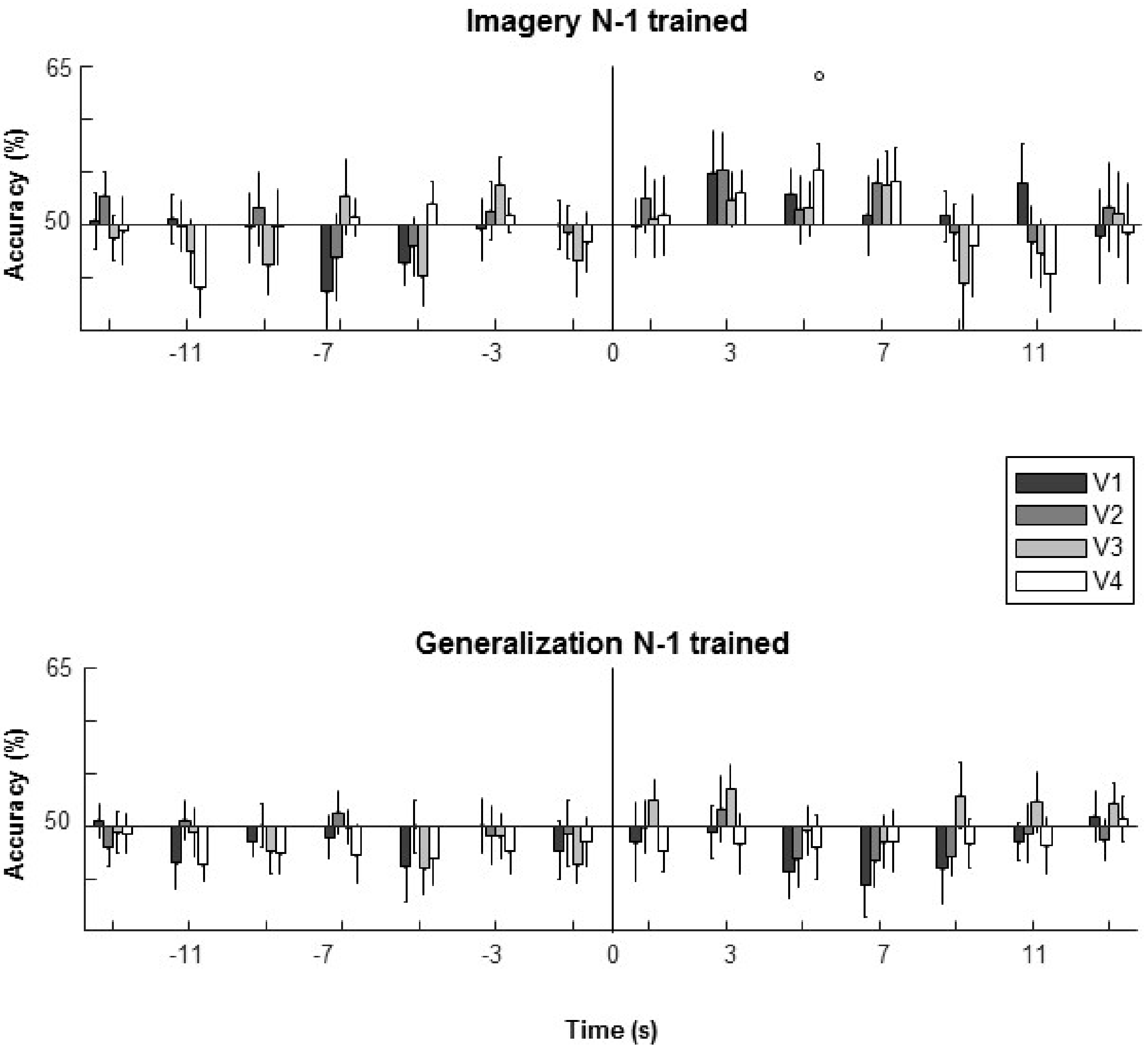

Figure S7. **ROI spillover effect control.** We conducted a control analysis to test whether our results could be explained by activity from the previous trial (spillover effect) on imagery and perception-imagery generalization (top and bottom panels, respectively). We thus trained and tested our classifiers on data with shifted labels (N-1). If there was a spill over from previous trial, this analysis should show higher decoding accuracy in the pre-imagery period compared to the original analysis (for details on the rationale see Materials and Methods section and Soon, Allefeld, Bogler, Heinzle, & Haynes, 2014). We found no significant above chance classification on the pre-imagery period but we did found significant decoding accuracy in V4 at +5s from imagery onset in the imagery condition. Nevertheless, this result indicates that effects in the pre-imagery time cannot be explained by activity spill over from previous trials. Error bars represent ±SEM across participants.

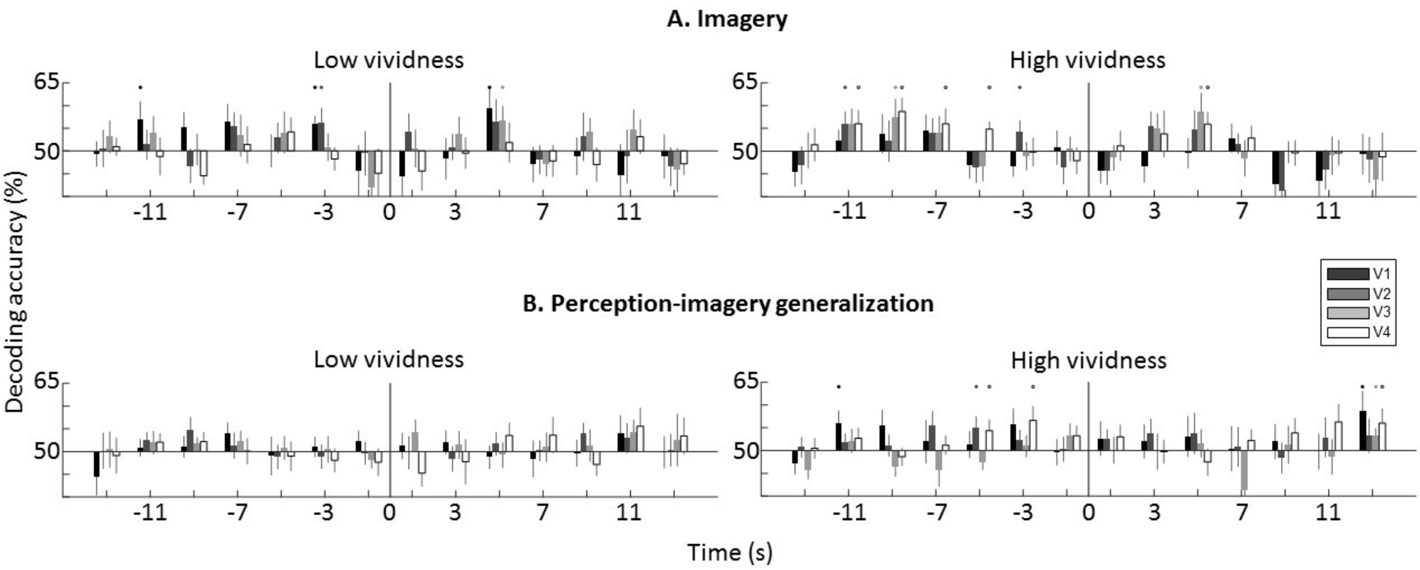

Figure S8**. Imagery-content decoding for low- and high-vividness trials.** We divided data into trials with low and high vividness (see text for details). High vividness trials showed higher decoding accuracy than low vividness trials. Greatest differences in decoding accuracy as a function of vividness were seen in the pre-imagery period, suggesting that subjective imagery vividness depends upon neural activity from before imagery. Error bars represent SEM across participants. Full points represent above chance decoding (p<0.05, one-tailed t-test).

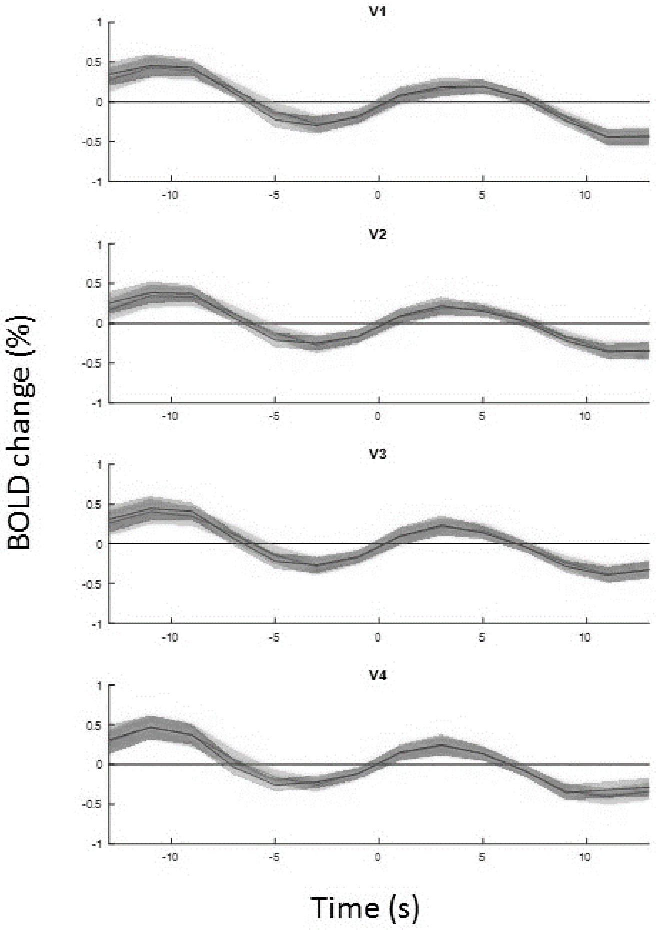

Figure S9. **BOLD amplitude change for different imagined gratings.** We tested if our effects could be explained by an overall amplitude differences between imagines gratings (i.e., univariate difference). We thus calculated the signal change for every voxel. Dark and light gray represent horizontal and vertical gratings, respectively. BOLD signal changes from both imagined gratings are overlapped and no significant differences were found (p>0.05, t-test, uncorrected). Note that the two peaks (at -10 and 4s) are likely to correspond in part to the changes occurring on the screen. The -10s peak is explained by the prompts "What did you imagine?" and "How vivid was it?" appearing on the screen at the end of the previous trial. The peak at 4s is in part explained by the engagement of the visual cortex in imagery and by the fixation point becoming brighter for 100ms to indicate the participants that the imagery onset time was recorded. Importantly, the perceptually evoked activities are uninformative about the contents of imagery, as the same prompts were shown irrespective of the chosen grating. Shade areas represent SEM.

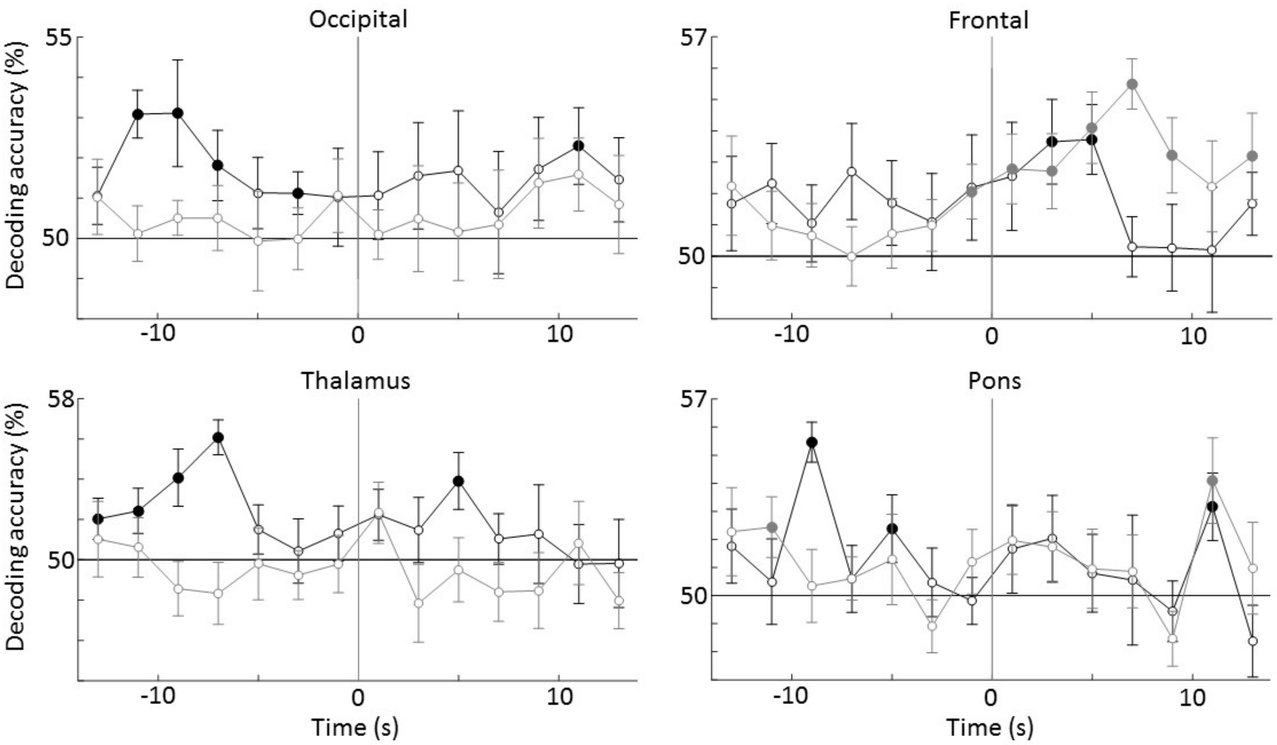

Figure S10. **Decoding on a subset of participants.** Based in a post-experiment interview we pinpoint some participants (n=4) that could not help thinking about gratings in some trials during the inter trial interval. In order to test if the effects reported here could be explained by these subset of participants, we performed the analysis on the remaining participants (n=10) who reported not having any thoughts or mental images about gratings during the inter trial interval. The control analysis revealed similar results to those presented on Figure 2, thus indicating that our results cannot be explained by the participants who had troubles keeping their minds free from gratings in the inter-trial period. Error bars represent SEM across participants. Solid circles represent significant above-chance decoding time-points ($p<0.05$, right-tailed t-test).

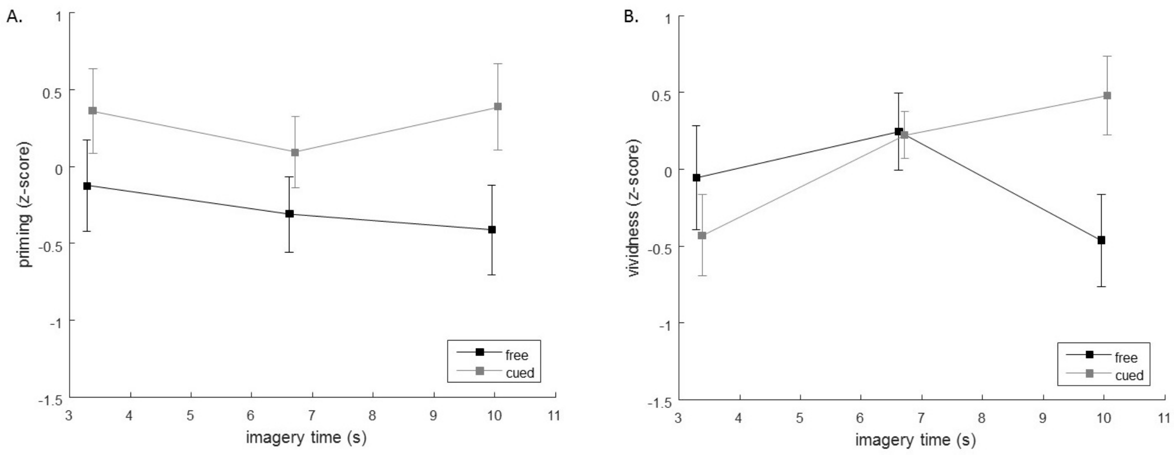

Figure S11. **Priming (A) and vividness (B) for a set of unexperienced participants (N=10) in the behavioral imagery onset reliability experiment.** While the main effect of condition (free vs cued) trended in the same way as for the experienced participants tested in the fMRI experiment (e.g., cued condition led to more priming than the free condition, Figure 3B-C), no significant effect was found. Importantly, we found no significant correlation between the imagining time and priming. This discrepancy with the results on figure 3 might be due to the fact that unexperienced participants were unable to holding a stable mental image during the imagery period.

	**A. MNI x,y,z (mm)**	**B. Atlas labelling**
**Occipital**	-17,-95,2	Middle occipital lobe (L) 36.1%<br>Calcarine (L) 27.9%<br>Superior occipital lobe (L) 14.3%<br>Inferior occipital lobe (L) 14.3%
**Frontal**	-36,32,18	Inferior frontal gyrus, triangular (L) 57.5%<br>Middle frontal gyrus (L) 6.6%
**Thalamus**	1,-7,5	Thalamus (R) 27.2%<br>Thalamus (L) 11.2%
**Pons**	7,-19,-24	Brainstem 34.8%<br>Cerebellum (R) 9.5%<br>Parahippocampus (R) 5.7%

Table S1. **Searchlight clusters locations.** A. Center of mass in MNI coordinates of the 4 clusters found in the searchlight analysis (occipital, frontal, thalamus and pons). B. Structure definitions according to the AAL atlas [5], except for the pons label which was defined using Freesurfer's subcortical automatic segmentation [6]. Note that Freesurfer's subcortical segmentation does not define subdivisions within the brainstem. We thus identified the pons by visual inspection of activations within the brainstem, which were mostly located in the brainstem's anterior protrusion rostral to the medulla, consistent with the pons location [7]. Percentages represent atlases' labels volume occupied by the clusters.

		Searchlight				ROI			
		F	O	P	T	V1	V2	V3	V4
Imagery	Decoding accuracy (%)	50.0092	50.0051	49.9879	49.9937	49.9861	49.9982	49.9646	49.9835
	Skewness	0.0658	0.0248	0.0439	0.0243	-0.05	-0.033	-0.0088	-0.0401
	Kurtosis	2.9579	2.9974	2.9668	2.9549	2.9106	2.9598	2.9714	2.9503
Generalization	Decoding accuracy (%)	49.9926	49.9985	50.0033	50.0079	50.0203	50.0457	49.9951	49.9533
	Skewness	-0.0514	-0.0135	0.0039	-0.0212	-0.0251	-0.0162	-0.0237	0.0046
	Kurtosis	3.0005	2.9663	2.9476	3.0354	2.9661	2.9996	3.0071	2.9486

Table S2. **Distribution of the empirically-determined decoding null-hypothesis.** We verified the normality of the null-hypothesis decoding distributions (determined using permutation tests, see text for details) by calculating the mean decoding accuracy, skewness and kurtosis. We calculated these values for each cluster in the searchlight analysis: fontal (F), occipital (O), pons (P) and thalamus (T) and each visual ROI: V1, V2, V3 and V4, for the imagery and generalization conditions. The expected decoding accuracy is 50% for the null hypothesis. Expected values of skewness are between -1 and 1; and kurtosis of 3 for normal distributions [8]. Our results show that decoding null-hypothesis distributions for both conditions (imagery and generalization) and decoding methods (searchlight and ROI) are centered on 50% and fulfill normal distributions criterion, thus validating the use of standard parametric statistical tests.

Supplementary References

1. Harrison, S. a & Tong, F. Decoding reveals the contents of visual working memory in early visual areas. *Nature* **458,** 632–635 (2009).
2. Reddy, L., Tsuchiya, N. & Serre, T. Reading the mind's eye: Decoding category information during mental imagery. *Neuroimage* **50,** 818–825 (2010).
3. Lee, S.-H., Kravitz, D. J. & Baker, C. I. Disentangling visual imagery and perception of real-world objects. *Neuroimage* **59,** 4064–4073 (2012).
4. Soon, C. S., Allefeld, C., Bogler, C., Heinzle, J. & Haynes, J. D. Predictive brain signals best predict upcoming and not previous choices. *Front. Psychol.* **5,** 1–3 (2014).
5. Tzourio-Mazoyer, N. *et al.* Automated Anatomical Labeling of Activations in SPM Using a Macroscopic Anatomical Parcellation of the MNI MRI Single-Subject Brain. *Neuroimage* **15,** 273–289 (2002).
6. Fischl, B. *et al.* Whole brain segmentation: automated labeling of neuroanatomical structures in the human brain. *Neuron* **33,** 341–55 (2002).
7. Pritchard., T. C. & Alloway, K. D. *Medical Neuroscience*. (Hayes Barton Press, 1999).
8. Bulmer, M. G. *Principles of Statistics*. (Dover Publications, 1979).
